# Highly diverse sputum microbiota correlates with the disease severity in patients with community-acquired pneumonia: a longitudinal cohort study

**DOI:** 10.1186/s12931-024-02821-2

**Published:** 2024-05-29

**Authors:** Jing Yang, Jinman Li, Linfeng Zhang, Zijie Shen, Yan Xiao, Guoliang Zhang, Mingwei Chen, Fuhui Chen, Ling Liu, Ying Wang, Lan Chen, Xinming Wang, Li Zhang, Lu Wang, Zhang Wang, Jianwei Wang, Mingkun Li, Lili Ren

**Affiliations:** 1 Beijing Institute of Genomics, Chinese Academy of Sciences, China National Center for Bioinformation, Beijing, 100101 China; 2 University of Chinese Academy of Sciences, Beijing, 100049 China; 3 Changping Laboratory, Beijing, 102206 China; 4 NHC Key Laboratory of Systems Biology of Pathogens and Christophe Mérieux Laboratory, National Institute of Pathogen Biology, Chinese Academy of Medical Sciences and Peking Union Medical College, Beijing, 100730 China; 5 Key Laboratory of Respiratory Disease Pathogenomics, Chinese Academy of Medical Sciences and Peking Union Medical College, Beijing, 100730 China; 6 State Key Laboratory of Respiratory Health and Multimorbidity, National Institute of Pathogen Biology, Chinese Academy of Medical Sciences and Peking Union Medical College, Beijing, 100730 China; 7 Shenzhen Third People’s Hospital, Shenzhen, 518112 China; 8 The First Affiliated Hospital of Xi’an Jiaotong University, Xi’an, 710061 China; 9 The Second Affiliated Hospital of Harbin Medical University, Harbin, 150001 China; 10 Jiangsu Provincial Key Laboratory of Critical Care Medicine, Department of Critical Care Medicine, School of Medicine, Zhongda Hospital, Southeast University, Nanjing, 210009 China; 11 Institute of Ecological Sciences, South China Normal University, Guangzhou, 510631 China

**Keywords:** Community-acquired pneumonia, Sputum microbiota, 16S rRNA, Longitudinal study, Disease severity

## Abstract

**Background:**

Community-acquired pneumonia (CAP) is a common and serious condition that can be caused by a variety of pathogens. However, much remains unknown about how these pathogens interact with the lower respiratory commensals, and whether any correlation exists between the dysbiosis of the lower respiratory microbiota and disease severity and prognosis.

**Methods:**

We conducted a retrospective cohort study to investigate the composition and dynamics of sputum microbiota in patients diagnosed with CAP. In total, 917 sputum specimens were collected consecutively from 350 CAP inpatients enrolled in six hospitals following admission. The V3-V4 region of the 16 S rRNA gene was then sequenced.

**Results:**

The sputum microbiota in 71% of the samples were predominately composed of respiratory commensals. Conversely, 15% of the samples demonstrated dominance by five opportunistic pathogens. Additionally, 5% of the samples exhibited sterility, resembling the composition of negative controls. Compared to non-severe CAP patients, severe cases exhibited a more disrupted sputum microbiota, characterized by the highly dominant presence of potential pathogens, greater deviation from a healthy state, more significant alterations during hospitalization, and sparser bacterial interactions. The sputum microbiota on admission demonstrated a moderate prediction of disease severity (AUC = 0.74). Furthermore, different pathogenic infections were associated with specific microbiota alterations. *Acinetobacter* and *Pseudomonas* were more abundant in influenza A infections, with *Acinetobacter* was also enriched in *Klebsiella pneumoniae* infections.

**Conclusion:**

Collectively, our study demonstrated that pneumonia may not consistently correlate with severe dysbiosis of the respiratory microbiota. Instead, the degree of microbiota dysbiosis was correlated with disease severity in CAP patients.

**Supplementary Information:**

The online version contains supplementary material available at 10.1186/s12931-024-02821-2.

## Introduction

Community-acquired pneumonia (CAP) is an acute respiratory infection acquired outside the hospital, affecting alveoli and distal airways, with variable symptoms including cough, fever, dyspnea, and expectoration [1]. The incidence of lower respiratory tract infection (LRI), which includes CAP, was 5,837 cases and 6,832 cases per 100,000 population among females and males, respectively [2]. It resulted in high morbidity and mortality rates in all age groups, especially in children and the elderly [2]. LRI remained the fourth leading cause of global years of life lost in 2019 before the COVID-19 pandemic [3]. 

Recent culture-independent studies revealed that the respiratory tract was not sterile in healthy individuals [4], and the lower respiratory tract microbiota contributed to the ecological and immunological homeostasis of the lung, influencing lung health and susceptibility to infections [5]. Although pathogen invasion is considered the cause of CAP, the causative agents are detected in fewer than 50% of CAP patients [4]. Studies have identified significant differences in the respiratory microbiota between CAP patients and healthy individuals, with the former being less diverse and enriched with pathogenic microbes such as *Pseudomonas*, *Staphylococcus*, and *Klebsiella* [6–9]. Additionally, the respiratory microbiota may influence pneumonia susceptibility via impeding colonization and immunological modulation [10, 11]. However, previous studies primarily focused on high-risk populations, such as human immunodeficiency virus (HIV) patients, lung transplant recipients, and children, and often with a small size of patients [6–8]. The association between respiratory microbiota and CAP in immunocompetent adults remains unclear. The interpretation is further complicated by diverse pathogens, the use of antibiotics, intubation, and corticosteroid therapies in CAP patients. Therefore, a comprehensive microbiota study in the general population, especially those untreated, is needed.

The respiratory microbiota is heterogeneous due to various host and environmental factors, including genetic background, mode of birth, feeding type, and inhaled pollutants [12–14]. Thus, clarifying the role of a specific variable in shaping the respiratory microbiota is challenging in cross-sectional studies. In contrast, longitudinal studies can pinpoint particular microbiota changes associated with a specific condition by controlling other covariates. Although longitudinal studies have been conducted on lung transplantation, chronic obstructive pulmonary disease (COPD), cystic fibrosis, COVID-19, and ventilator-associated pneumonia [15–19], studies on the lower respiratory microbiota in CAP patients are limited. Studying microbiota changes during the disease process will provide insights into the role of the respiratory microbiota in disease development.

In this study, we collected time-series sputum samples from CAP patients starting from the first day after admission, prior to therapy administration. We identified a correlation between the composition and dynamics of the sputum microbiota and disease severity, revealing distinct microbiota compositions in patients with different pathogens. This suggests that the dysbiosis of the sputum microbiota could potentially serve as a valuable diagnostic and prognostic marker for pneumonia. Furthermore, it presents a possible target for intervention in the management of the condition.

## Results

### Overview of the samples and sequencing data

**Fig. 1:**
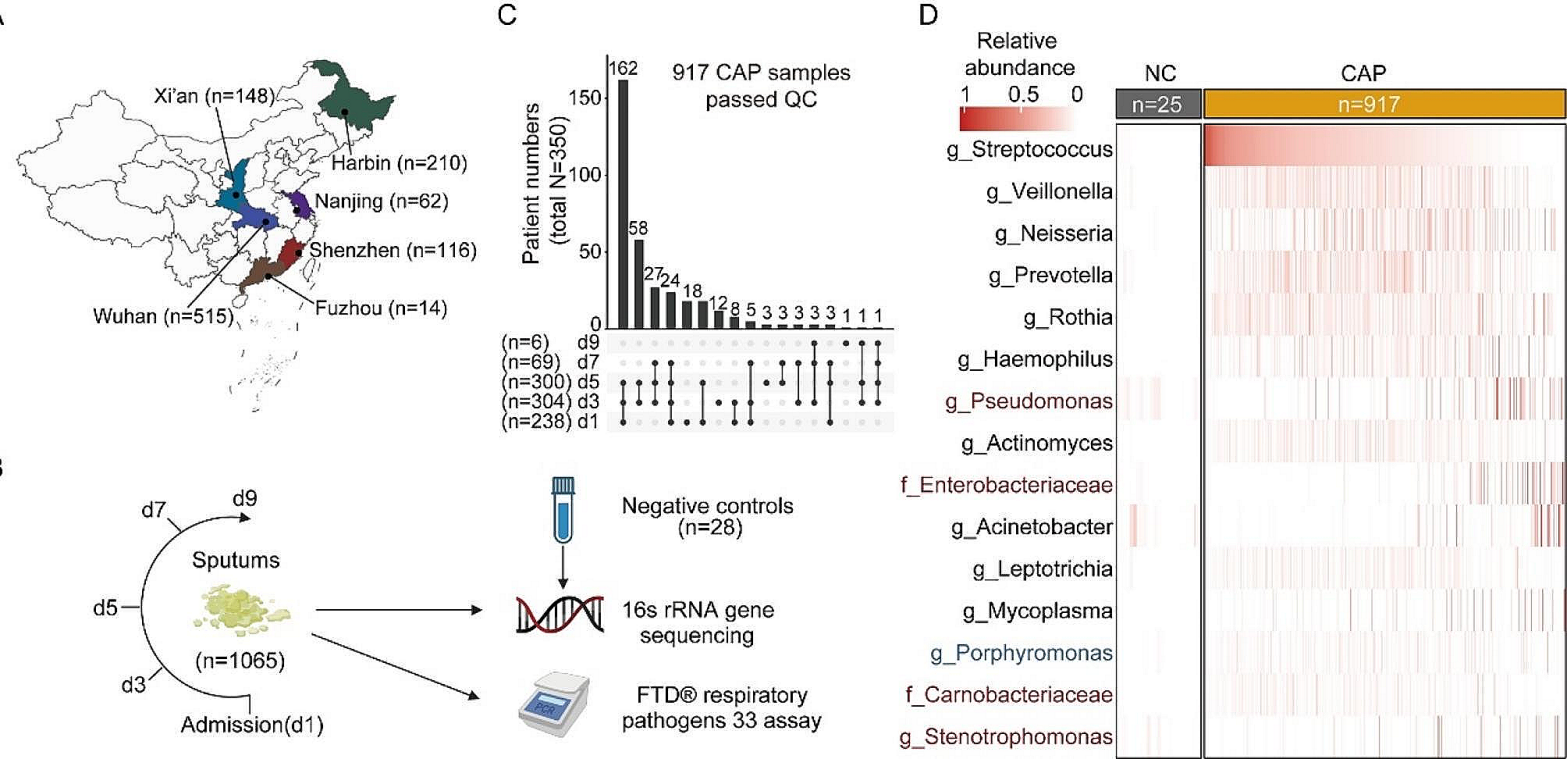
**Study design and sputum microbiota composition.** (**A**) Geographic distribution of the samples. n: sample size. (**B**) Sampling strategy. d: days after admission. (**C**) Summary of the collected samples. (**D**) Abundance of bacteria in CAP patients and negative controls. The top 15 bacteria with the highest average relative abundance in CAP patients are shown. Bacteria that are more enriched in CAP patients than in all three HCs are labeled in red, while those enriched in HCs are labeled in blue

Longitudinal sputum samples (1,065) were collected from 367 inpatients diagnosed with CAP in six hospitals across representative geographical locations in China. Following quality filtering, 917 samples from 350 patients and 25 negative controls (NCs) were used for subsequent analysis (Fig. 1A-C). The composition of sputum microbiota of CAP patients was notably different from that of NCs (PERMANOVA, R^2^ = 0.2, *p* = 0.001, Fig. S1A), and the five most abundant taxa in NCs (*Sphingomonas*, *Blastomonas*, *Methylobacterium*, *Bosea*, and *Propionibacterium*, Fig. S1B) comprised 53.1% of all NCs sequence, while comprising 2.7% of all sequence in CAP samples, indicating minimal background contamination.

The median days from symptoms onset to admission were six (IQR 3–7). Forty-one (12.0%) patients had chronic pulmonary diseases, including COPD, asthma, and bronchiectasis. Before admission, 66 (19.5%) patients took antibiotics within five days, and 15 (5.5%) patients used immunosuppressants. Fifty-five (16.3%) patients were diagnosed with severe cases and seven of them died. Notably, five clinical severity indicators, including the use of invasive mechanical ventilation, CURB65 scores, pneumonia severity index (PSI) scores, duration of oxygen supplementation, and length of hospital stay, were all significantly higher in severe cases than in non-severe cases (Fisher’s exact test or Wilcoxon signed-rank test, *p* < 0.05). More demographic and clinical information was provided in Table 1 and Table S1. Meanwhile, 876 sputum microbiotas in Chinese healthy individuals (with no acute or chronic respiratory diseases) from three previous studies were used as the healthy controls (HCs) in the study (Table S2 and S3) [20–22]. 

**Table 1:** Metadata of study subjects and their correlations with the sputum microbiota on admission

	All CAP subjects (*n* = 350)		CAP subjects with day1 samples (*n* = 238)			
Variables	Median (IQR)/ *n* (%)	No. of subjects	Median (IQR)/ *n* (%)	No. of subjects	*R*^2^ (uni)^c^	*R*^2^ (multi)^d^
1. Age	58 (41–69)	303	58(43–70)	211	0.038**	
2. Sex, male	207(66.6)	311	155(69.2)	224	0.003	
3. BMI	22.5 (20.6–24.5)	339	22.5(20.3–24.5)	230	0.001	
4. Current smoker	94 (32.0)	294	70(32.7)	214	0.002	
5. Chronic pulmonary disease^a^	41 (12.0)	341	33(14.3)	231	0.005	
6. Antibiotic use (before admission),	66 (19.5)	338	48(20.9)	230	0.004	
7. Immunosuppressant use (before admission)	15 (5.5)	275	7(3.5)	200	0.008	
8. Days between onset and admission	6 (3–7)	284	7 (4–7)	192	0.009	
9. City						
Wuhan	161 (46.0)		142 (59.7)			
Harbin	70 (20.0)		26 (10.9)			
Xi’an	48(13.7)	350	42 (17.7)	238	0.138**	
Shenzhen	44 (12.6)		15 (6.3)			
Nanjing	22 (6.3)		10 (4.2)			
Fuzhou	5 (1.4)		3 (1.3)			
10. Possible pathogens^b^						
Bacteria	90 (39.6)	227	58 (36.0)	161	0.043	
Viruses	88 (38.8)		67 (41.6)			
Mix	38 (16.7)		26 (16.1)			
11. Severity, severe	55 (16.3)	337	39(17.0)	230	0.030**	0.012*
12. Invasive mechanical ventilation	18 (5.7)	314	13(5.7)	227	0.011**	0.009
13. CURB65 score						
0	170 (49.9)		108 (46.6)			
1	118 (34.6)	341	82 (35.3)	232	0.021**	0.018*
2	44 (12.9)		35 (15.1)			
3	7 (2.1)		6 (2.6)			
4	2 (0.1)		1 (0.4)			
14. PSI score	58.5 (33–82)	338	64 (41.5–84)	231	0.012*	0.016*
15. Duration of oxygen supplementation	1 (0–9)	305	3.5(0-9.25)	220	0.021**	0.033*
16. Length of hospital stay	11 (8–16)	130	10.5 (8–15)	68	0.015*	0.022*
17. Clinical outcome, death	7 (2.1)	339	7(3.0)	230	0.023*	0.021*

^a ^Chronic pulmonary disease includes COPD, asthma, and bronchiectasis;

^b ^The pathogens identified through the FTD assay in the initial positive sample collected within three days after admission for each subject. Mix represents bacterial-viral coinfection;

^c ^R^2^ (uni) denotes the proportion of variance explained by the variable in the univariate PERMANOVA analysis of 238 CAP sputum samples on admission

^d ^R^2^ (multi) denotes the proportion of variance explained by each patient’s clinical status indicators (variables 11–17) in the multivariate PERMANOVA analysis of 238 CAP sputum samples on admission, adjusted for all the possible confounders (variables 1–10)

* p.adj < 0.05, ** p.adj < 0.01, *** p.adj < 0.001

### Sputum microbiota composition in CAP patients

Six commensal microbes that are frequently observed in the respiratory tract, including *Streptococcus*, *Veillonella*, *Neisseria*, *Prevotella*, *Rothia*, and *Haemophilus*, showed the highest relative abundance and accounted for 51.2% of CAP microbial reads, 38.0% of HC microbial reads, and 1.4% in NCs (Fig. 1D). The sputum microbiota diversity and composition in CAP patients were significantly different from HCs (PERMANOVA, mean R^2^ = 0.13, *p* < 0.001; Fig. S1A). Possible pathogens, including *Pseudomonas*, *Enterobacteriaceae*, *Sphingomonas*, and *Stenotrophomonas*, were significantly enriched in CAP samples compared to all three HC populations (Fig. S1C).

**Fig. 2:**
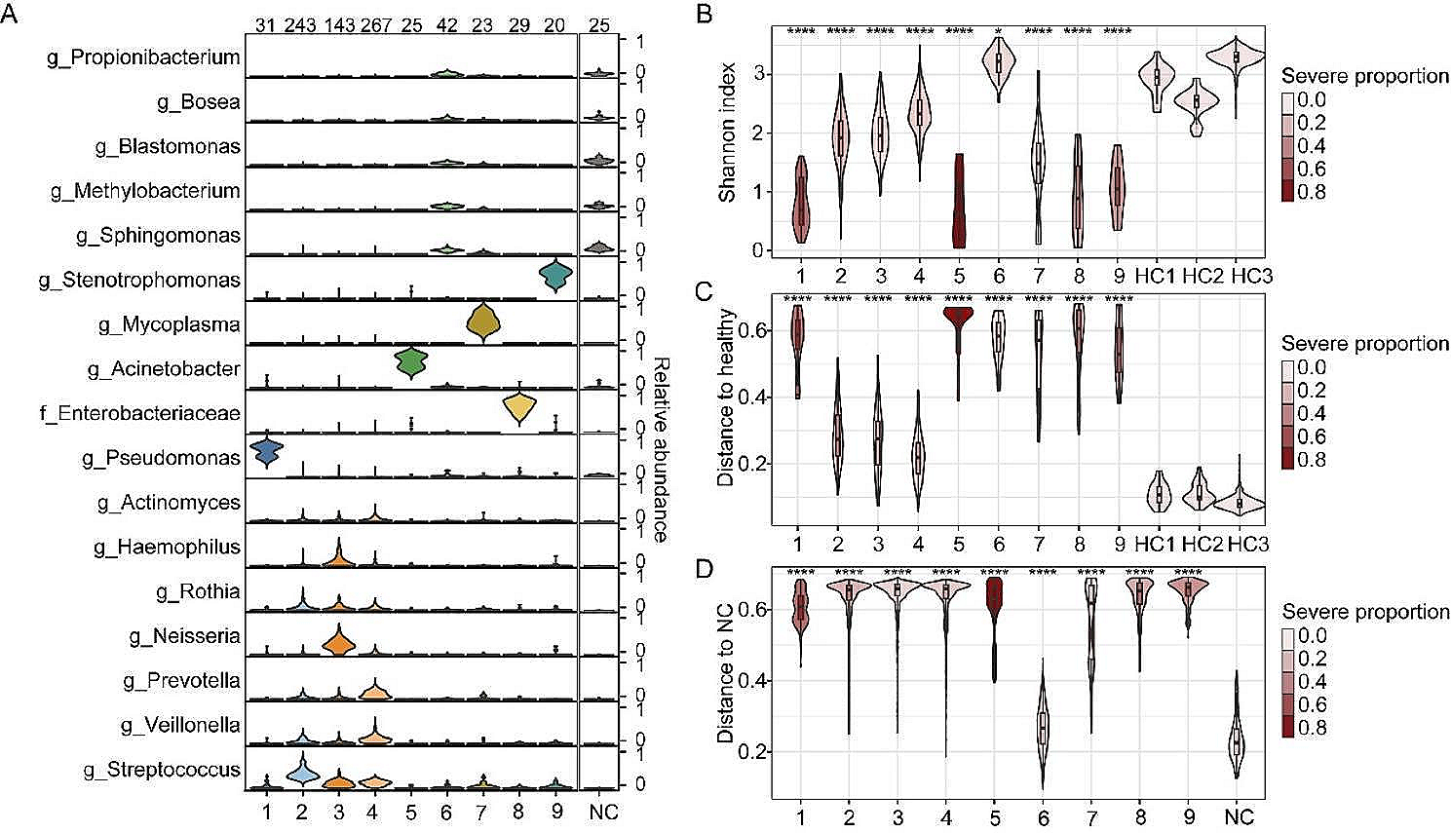
**The composition of sputum microbiota clusters in CAP patients.** (**A**) The compositions of bacteria in different clusters. Bacteria with average relative abundances greater than 5% in at least one cluster are shown. The sample numbers in each cluster and NCs were labeled above the figure. (**B**) Shannon index of each cluster and HCs. (**C**) JSD distance between different CAP clusters and healthy individuals. The microbiota composition of the three HC groups was averaged and used as the HC to calculate the distance. (**D**) JSD distance between different CAP clusters and NCs. In B and C, statistical significance was determined by comparing each cluster with all the HCs, and the color of the plot in B, C, and D denotes the proportion of severe cases in each CAP cluster. * *p* < 0.05, ** *p* < 0.01, *** *p* < 0.001, **** *p* < 0.0001

The major component of sputum microbiotas in CAP patients showed significant heterogeneity among different individuals (Fig. 1D). Employing clustering algorithms on the microbiota data revealed the presence of nine distinct clusters (CSs) (Fig. 2A, Fig. S2A). The robustness of these clusters was confirmed by bootstrap analysis (Fig. S2B, mean Rand index = 0.85, see Supplementary methods). These CSs could be further classified into three microbiota types: CS2, CS3, and CS4 (CS2-4), which were found in 71.1% of CAP patients, exhibited higher alpha diversity than other clusters, except for CS6 (Fig. 2B). CS2-4 were dominated by commensal bacteria (Fig. 2A) and showed higher similarity to healthy controls (Fig. 2C). In contrast, CS1, CS5, and CS7-9 (CS1,5,7,8,9) were dominated by possible pathogens. They had lower alpha diversity, higher dominant bacteria abundance, and were more distinct from HCs compared to CS2-4 (Fig. 2B-C, Fig. S2C). Microbiotas in CS6 exhibited the highest similarity to NCs and were more prevalent in specific individuals than randomly distributed (Fig. 2D, Fig. S2D-F), suggesting that the sputum samples of CS6 were either relatively sterile or challenging to collect. Additionally, CS6 did not appear to be detected more frequently in the later period of hospitalization, suggesting no association with post-admission treatment (Fig. S2G). Notably, the severity rate (incidence of severe condition) in CS2-4 patients was 12.9%, similar to CS6 (4.7%, Fisher’s exact test, *p* = 0.213), but significantly lower than CS1,5,7,8,9 (36.4%, Fisher’s exact test, *p* < 0.001).

### Association between sputum microbiota and disease severity

We then investigated the association between clinical and demographic features and the sputum microbiota. To minimize the impact of antibiotic use and other medical interventions after admission, only 238 samples collected on the first day after admission were used for the subsequent analyses. We found that disease severity (diagnosed by the clinician, see Methods), as well as five clinical indicators that correlated with disease severity, including CURB65 scores, PSI scores, duration of oxygen supplementation, length of hospital stay, and clinical outcome, were all significantly correlated with the sputum microbiota, after controlling for the possible confounders (Table 1, confounders: variables 1–10). In individual geographic sites, the correlation with disease severity remained statistically significant in Wuhan, with the largest sample size (*n* = 142, PERMANOVA, R^2^ = 0.021, *p* < 0.05), suggesting that the correlation was not influenced by the differences in patient enrollment across various geographic locations. Notably, the use of antibiotics and immunosuppressive drugs before admission, as well as days from onset, showed no significant impact on the microbiota composition (Table 1). This might be attributed to the limited sample size in this study. Nevertheless, variables 1–10 in Table 1 were all included as covariates whenever applicable in subsequent multivariate analyses.

**Fig. 3:**
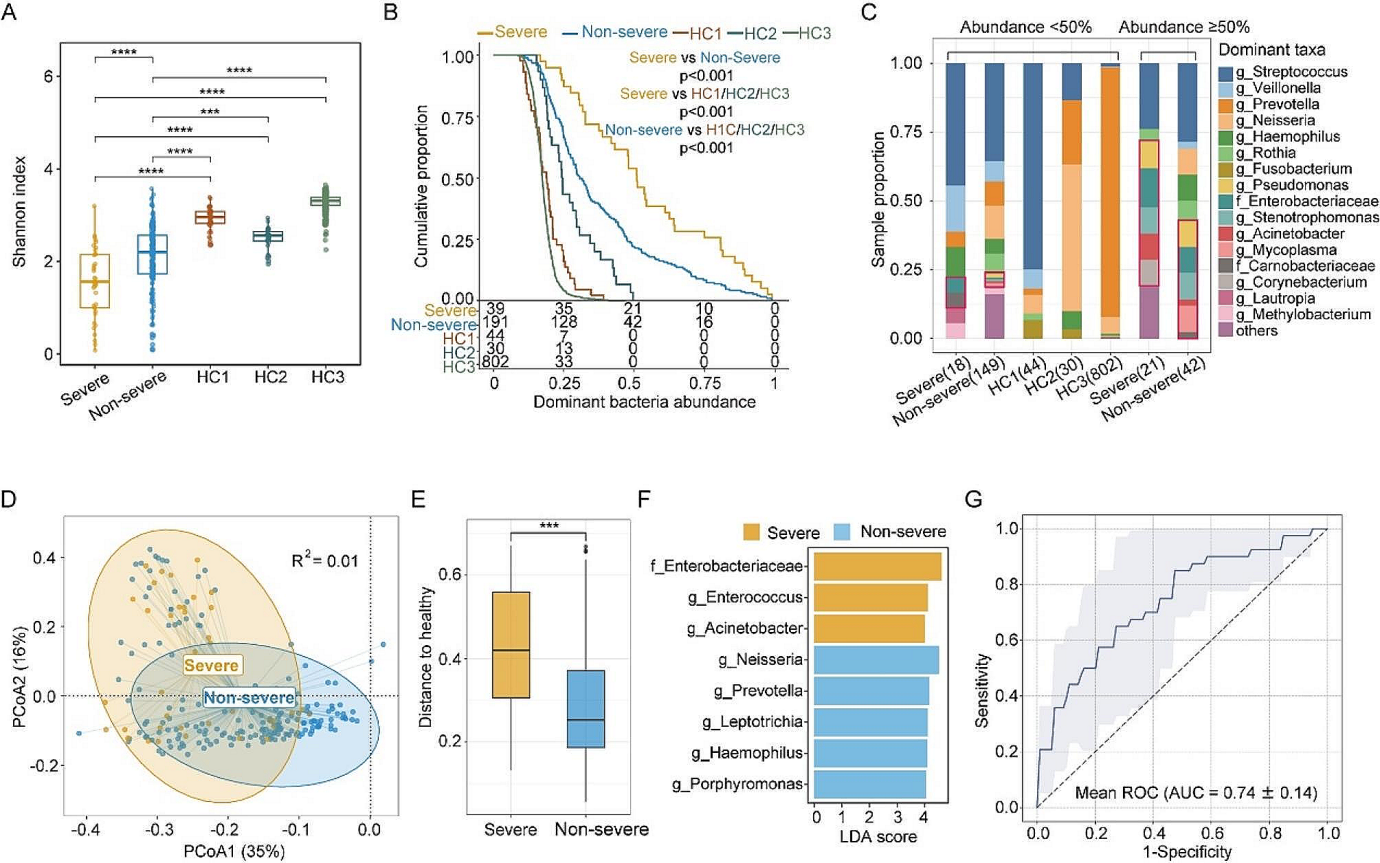
**Difference in the sputum microbiota between CAP patients with varying degrees of severity and healthy individuals.** (**A**) Shannon index of the microbiota of CAP patients on admission and healthy individuals. (**B**) Distribution of the abundance of the predominant bacterium in CAP patients and HCs. The y-axis indicates the proportion of patients with a dominating bacterium whose abundance is greater than that indicated on the x-axis. The number of patients with a dominating bacterium whose abundance is greater than 0, 25%, 50%, 75%, and 100% is shown below the x-axis. The p-value was calculated by log-rank test. (**C**) Proportion of dominant bacteria in CAP patients and HCs. Bacterial genera and families containing at least one known (opportunistic) pathogen, are highlighted with red boxes. The numbers in brackets on the x-axis indicate the number of samples. A list of pathogenic bacteria is provided in Table S4. Samples with dominating bacterium abundance higher than 50% and lower than 50% were shown separately. (**D**) PCoA plot of samples from severe and non-severe patients based on the JSD distance. R^2^ was calculated by PERMONAVA analysis. (**E**) JSD distance to healthy individuals of severe and non-severe CAP patients. The microbiota composition of the three HC groups was averaged and used as the HC to calculate the distance. (**F**) Bacteria correlated with the disease severity identified by LEfSe (LDA score > 4, *p* < 0.05). (**G**) ROC curve for the disease severity classifier based on the L1 regularized logistic regression model. * *p* < 0.05, ** *p* < 0.01, *** *p* < 0.001, **** *p* < 0.0001

First, we found that the alpha diversity of sputum microbiotas in non-severe patients was less deviated from the three healthy cohorts compared to severe patients (Fig. 3A). Moreover, the microbiotas in 53.8% of severe patients were dominated by bacteria with abundances greater than 50%, whereas the fraction was 22.0% and 0% in non-severe patients and healthy individuals, respectively (Fig. 3B). Additionally, dominant bacteria with abundances greater than 50% comprised more possible pathogens, especially in severe patients (Fig. 3C).

The composition of sputum microbiota differed considerably between severe and non-severe patients (multivariate PERMANOVA R^2^ = 0.02, *p* < 0.05; Fig. 3D), which were both distinct from healthy individuals (PERMANOVA, *p* < 0.001; Fig. S3A). Microbiotas in severe patients were more disrupted relative to healthy individuals than those in non-severe patients (Fig. 3E). LEfSe analysis revealed increased abundance of possible pathogens, including *Enterobacteriaceae*, *Acinetobacter*, and *Enterococcus* in severe cases, while commensal bacteria, including *Haemophilus*, *Neisseria*, and *Prevotella* were more abundant in non-severe cases (*p* < 0.05, Fig. 3F). MaAsLin2 analysis identified enrichment of *Enterobacteriaceae* in severe cases, and its abundance was also positively correlated with the duration of oxygen supplementation, while adjusting for covariates (Fig. S3B). Additionally, a classifier utilizing the L1 regularized logistic regression model could distinguish the severe cases from non-severe cases using microbiota with moderate accuracy (AUC = 0.74; Fig. 3G). Key features selected for identifying severe cases included high abundances of *Enterobacteriaceae* and *Corynebacterium*, along with a low abundance of *Neisseria*. Furthermore, the analysis of patients from individual cities confirmed the enrichment of *Enterobacteriaceae* in severe patients (in Wuhan), suggesting that the identified signature was not an artifact due to variations in the patients enrolled from different cities.

The functional potential of the sputum microbiota was predicted using PICRUSt analysis [23]. Five of the top 10 pathways enriched in the severe cases (MaAsLin2 analysis) were related to menaquinol biosynthesis (Fig. S3C and D), with all five pathways contributed by *Enterobacteriaceae*. Menaquinones are involved in the post-translational modifications of proteins needed for blood coagulation [24], and their dysfunction has been proposed as a risk factor for the severity of CAP [25, 26]. Meanwhile, four pathways involving the fermentation of butanoate, primarily contributed by *Porphyromonas* and *Fusobacteria*, were enriched in non-severe cases (Fig. S3E). Butanoate has been shown to enhance T cell proliferation and activation while suppressing inflammatory reactions [27, 28]. 

### Dynamics of the sputum microbiota and its association with the disease severity

**Fig. 4:**
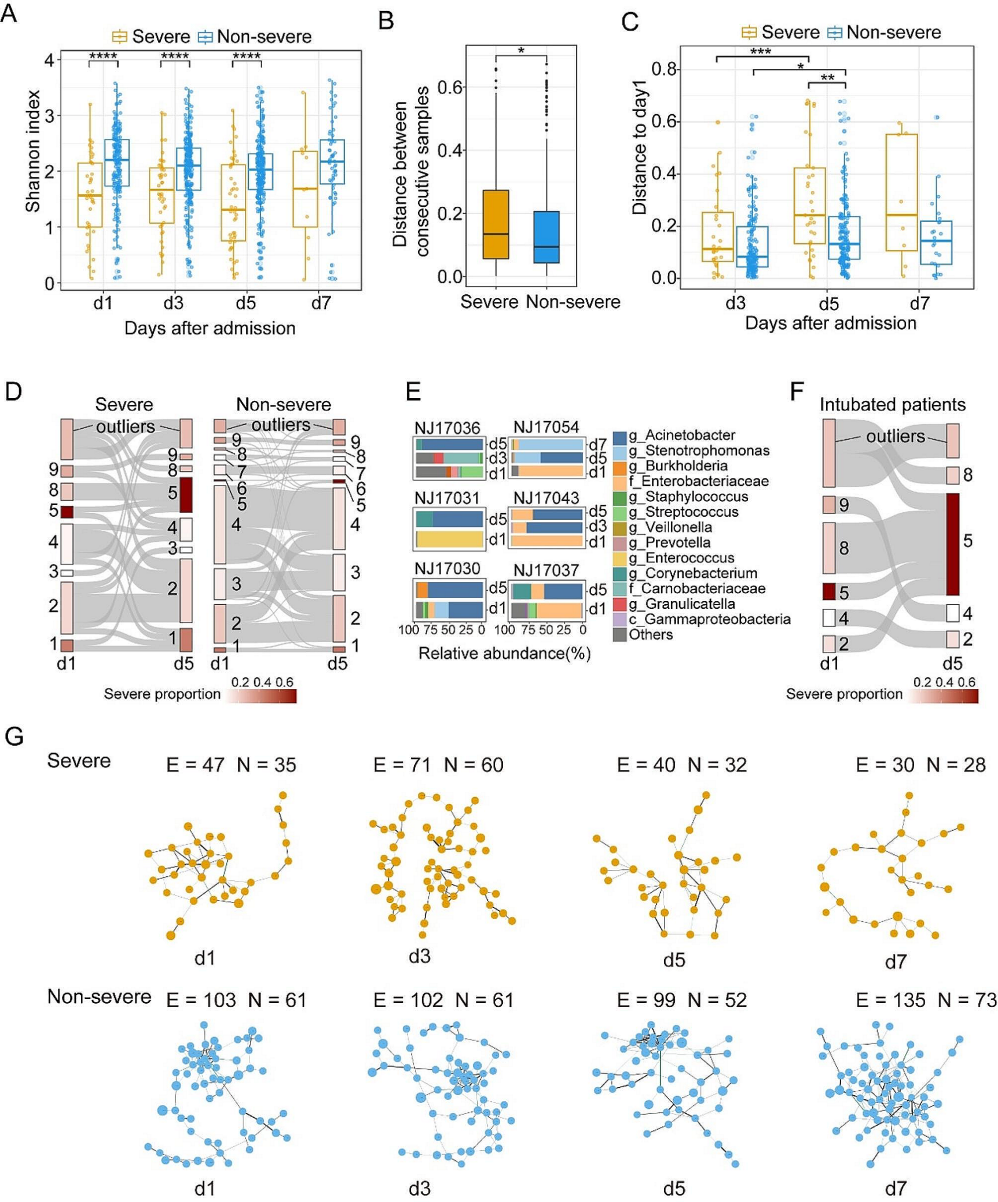
**Dynamics of the sputum microbiota and its association with disease severity.** (**A**) Shannon index of sputum microbiota in severe cases and non-severe cases at different time points after admission. (**B**) Differences in JSD distance between two consecutive samples from severe and non-severe cases. (**C**) JSD distance between samples on admission and samples collected at different sampling time points. (**D**) The transitional Sankey diagram of different microbiota clusters from day1 to day5 in severe and non-severe CAP cases. Outliers are samples that could not be assigned to any of the nine clusters. (**E**) The microbiota composition of six severe patients whose microbiota belonged to CS5 on day5. (**F**) The transitional Sankey diagram of microbiota clusters in 11 patients underwent invasive mechanical ventilation from day1 to day5. (**G**) Giant component of concurrent networks constructed by SpiecEasi in severe and non-severe CAP cases at different time points. Each node denotes a bacterial microbe and the size of nodes represents the mean abundance of microbes. Black lines represent positive correlations between microbes while green lines represent negative correlations. The thickness of the lines denotes the magnitude of the correlation. The number of edges (E) and nodes (N) are shown in the Figure. The same networks with microbial labels of nodes were shown in Fig. S4B. * p.adj < 0.05, ** p.adj < 0.01, *** p.adj < 0.001, **** p.adj < 0.0001

We further investigated how microbiota dynamics varied between non-severe cases and severe cases. First, the alpha diversity of microbiota in non-severe cases was significantly higher than that in severe cases at the first three time points (Fig. 4A), with no significant difference observed between different time points within the same group. Second, severe cases showed a larger longitudinal change in the microbiota composition (Fig. 4B), becoming more deviated from the initial state during hospitalization (Fig. 4C). Third, neither the severe nor the non-severe patients’ sputum microbiota altered toward a healthy state during hospitalization (Fig. S4A).

Then, we explored the CS transition pattern between Day 1 and Day 5, which encompassed the largest number of sample pairs (33 severe cases and 172 non-severe cases). First, cluster switching occurred more frequently in severe cases (66.7% vs. 43.6%, Fisher’s exact test, *p* < 0.05, Fig. 4D). Furthermore, transmissions between different CSs were likely non-random, as all three CS8 samples on Day 1 switched to CS5 on Day 5 in severe cases, whereas other CSs were rarely transmitted to CS5 (100% vs. 10.5%, Fisher’s exact test, *p* < 0.01, Fig. 4D). Specifically, all those three CS8 samples were dominated by *Enterobacteriaceae* (abundance > 62.8%) on Day 1, with abundance decreasing to less than 28.1% on Day 5, while *Acinetobacter* increased from less than 1.2% to more than 54% (NJ17037, NJ17043, and NJ17054 in Fig. 4E). Besides, all six severe patients with CS5 microbiota on Day 5 received invasive mechanical ventilation during hospitalization, suggesting that the expansion of *Acinetobacter* might be associated with secondary infection following the use of invasive mechanical ventilation. However, not all intubated patients transmitted to CS5 (6 out of 11; Fig. 4F) despite that the probability is much higher than that in non-intubated patients (54.6% vs. 2.1%, Fisher’s exact test, *p* < 0.01).

To explore the association between the dynamics of microbial interaction and disease severity in CAP, correlation networks were constructed for samples collected at different time points and in different groups. We found that the interactions between bacteria were remarkably sparser (with a small number of edges and degrees in the network) in severe patients than in non-severe patients at all time points (Fig. 4G, Fig. S4B). Meanwhile, we noted that the network contained more potential pathogens, such as *Enterobacteriaceae*, in severe patients compared to non-severe patients and HCs (Fig. S4B and C), suggesting a possible dysbiotic state of the sputum microbiota in severe patients. Furthermore, the number of network connections in the severe group decreased markedly but remained unchanged in the non-severe group, indicating that the sputum microbiota in severe patients may become more disordered during hospitalization.

### Sputum microbiotas varied between patients infected by different pathogens

**Fig. 5:**
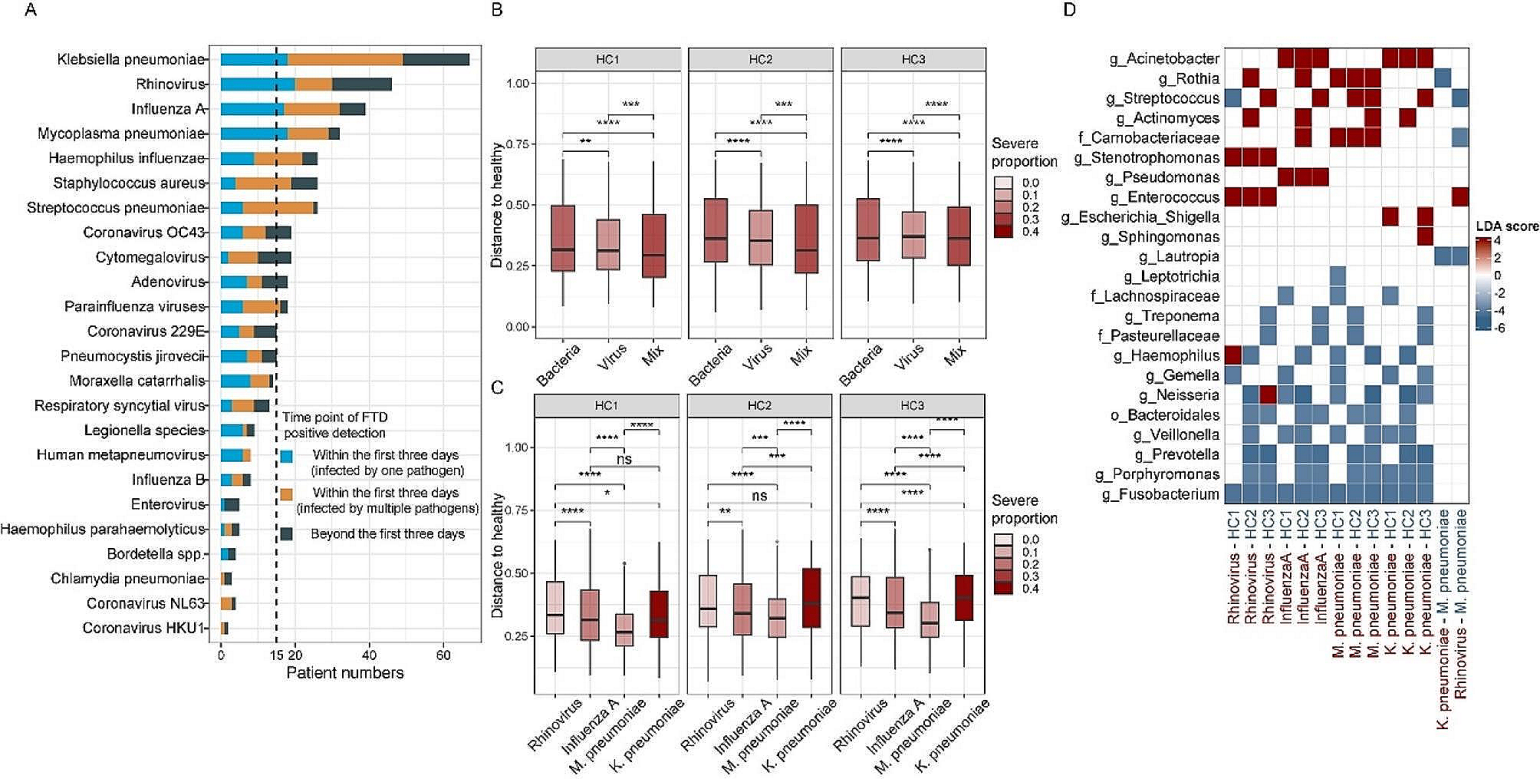
**Microbiota features in patients infected with different pathogens.** (**A**) Number of patients infected by different pathogens. (**B**) JSD distance to HCs for patients infected by bacteria, viruses, and mixed infection. (**C**) JSD distance to HCs for samples infected by Rhinovirus, Influenza A, *Mycoplasma pneumoniae*, and *Klebsiella pneumoniae*. (**D**) Bacteria that associated with different types of infections identified by LEfSe (|LDA| score > 4, *p* < 0.05). The LDA score denotes the extent of enrichment of the bacterium in the infection type that is labeled in red on the x-axis. * *p* < 0.05, ** *p* < 0.01, *** *p* < 0.001, **** *p* < 0.0001

Possible pathogens were identified in 548 samples from 256 patients by the FTD® Respiratory Pathogens 33 assay (Fig. 5A). Notably, there was good consistency between the result of 16 S rRNA gene sequencing and the FTD assay (Fig. S5A). To avoid secondary infection, only 216 patients with a positive FTD result within the first three days after admission were used in subsequent analyses (11 patients positive for *Pneumocystis jirovecii* were excluded due to the small sample size). Ninety patients were suspected to be infected by at least one bacterial pathogen, 88 patients were suspected to be infected by viruses, and Thirty-eight patients were coinfected by both bacterial and viral pathogens (mix). We observed a significant difference in the microbiota composition between bacterial and viral infections, as well as between viral and mixed infections, and microbiotas under the three conditions were all different from that in HCs (PERMANOVA, *p* < 0.05, Fig. S5B), with the bacterial infection samples showed greater deviations (Fig. 5B). Different bacteria were enriched in three distinct types of infections, whereas some commensal bacteria, such as *Fusobacterium*, were significantly depleted in all three types (Fig. S5C).

We then classified the infections into subgroups based on the pathogen detected, considering only those infecting more than fifteen patients (Rhinovirus, *Mycoplasma pneumoniae*, *Klebsiella pneumoniae*, and Influenza A) after excluding coinfection samples. Out of 18 patients detected with *Mycoplasma pneumoniae*, only three exhibited a predominance of *Mycoplasma* in their sputum microbiota (CS7, median *Mycoplasma* abundance = 42.4%), while the remaining samples were dominated by respiratory commensals (14 from CS2-4, one dominated by *Lautropia*, median *Mycoplasma* abundance = 2.6%). Similarly, only one of the 18 *Klebsiella pneumoniae*-positive patients was assigned to *Enterobacteriaceae*-dominant CS5, indicating that the pathogen was not obligatory as the predominant bacterium. The microbiota composition (excluding the pathogen itself) in all four infections differed from that in HCs (PERMANOVA, *p* < 0.05, Fig. S5D). Although no significant difference in alpha diversity was observed between patients infected with different pathogens (Fig. S5E), the microbiota alterations relative to the HCs in *Mycoplasma pneumoniae* infections was less significant than in other infections (Fig. 5C). Specifically, we noted that rhinovirus infections were enriched with *Enterococcus* and *Stenotrophomonas*, influenza A infections with *Acinetobacter* and *Pseudomonas*, *Mycoplasma pneumoniae* with *Rothia* and *Carnobacteriaceae*, while Acinetobacter was enriched in *Klebsiella pneumoniae* infections (Fig. 5D). The microbiota composition differed between *Mycoplasma pneumoniae* infections and the *Klebsiella pneumoniae* infections, as well as between *Mycoplasma pneumoniae* infections and rhinovirus infections (PERMANOVA, R^2^ = 0.0.81 and 0.078, *p* < 0.05, Fig. S5D).

## Discussion

Recent studies proposed that respiratory microbiota dysbiosis, especially low community diversity, was implicated in pneumonia development [9, 19]. However, due to the small sample size and the underrepresentation of immunocompetent patients in previous studies, characteristics of the lower respiratory microbiota in CAP patients remain largely unknown. In this study, we revealed key features of sputum microbiota in 350 CAP patients through an examination of 917 longitudinal sputum samples.

The sputum microbiota in CAP patients is highly diverse. In contrast to previous studies that identified a limited number of microbiota community types in healthy populations and patients with pneumonia or other pulmonary diseases [7, 29, 30], we identified a more heterogeneous microbiota composition in CAP patients in this study, with nine distinct microbiota clusters being identified, which may be attributed to a larger sample size, better sample representation, and diverse pathogen types. The commensal bacteria that are typically found in the respiratory tract of healthy populations make up the majority of the sputum microbiota in most patients, suggesting potential resistance or resilience of the respiratory microbiota against acute infection. Meanwhile, a sizeable proportion of samples (14.0%) had microbiota with unusually high abundances of possible pathogens, including *Enterobacteriaceae*, *Pseudomonas, Acinetobacter, Mycoplasma*, and *Stenotrophomonas*, all previously proposed as pneumonia-causing pathogens [19, 31–34], suggesting abnormal pathogen growth. In addition, 10.3% of samples had a microbiota predominated by non-typical pathogenic bacteria, such as *Corynebacterium*, *Rothia*, and *Haemophilus*, highlighting the complexity of the CAP microbiota (Fig. S2I). A special group of patients with a relatively sterile microbial community was also identified, a phenomenon previously observed in the bronchoalveolar lavage fluid of healthy individuals and COPD patients [5, 29]. However, the presence of such a low microbial load in CAP patients is unexpected, given that CAP is typically associated with the proliferation of invasive or colonized bacteria, triggering an inflammatory response [5, 35]. The severity rate of those patients was similar to that of commensals-dominated patients (CS2-4), and lower than that of the patients dominated by possible pathogens (CS1,5,7,8,9). We hypothesize that a stronger immune response or lack of sufficient resources might have suppressed the growth of both commensal and pathogenic microbes in these patients.

Second, the degree of sputum microbiota dysbiosis correlated with disease severity in CAP patients. In line with previous findings, severe CAP patients exhibited lower alpha diversity compared to healthy controls upon admission [6, 8, 9]. However, we noticed that the sputum microbiota in non-severe cases had alpha diversity less deviated from healthy controls, despite that their microbiota was still more likely to be predominated by a specific bacterium. Meanwhile, their microbiota compositions were more similar to those of healthy controls compared to severe cases. The most significant enriched bacterium in the sputum of severe cases is *Enterobacteriaceae*, commonly found in the gastrointestinal tract [36]. This increase may be due to the growth of colonizing bacteria or the translocation of gut bacteria to the respiratory tract, triggering systemic inflammation [37]. Furthermore, we observed a high transition rate from an *Enterobacteriaceae-*dominant microbiota to an *Acinetobacter*-dominant microbiota post-mechanical ventilation, suggesting increased vulnerability to ventilation-induced secondary infection in *Enterobacteriaceae-*dominant cases. Thus, a high level of *Enterobacteriaceae* in the sputum seems to predict a poor prognosis in CAP patients.

Third, the sputum microbiota in severe cases was more vulnerable and susceptible to significant changes during hospitalization, evidenced by higher compositional alteration, more frequent cluster switching, and more significant changes in the microbial network. This pattern resembles observations in other respiratory diseases like COPD and COVID-19 [16, 38], potentially influenced by both medical intervention and disease progression [12, 19]. However, distinguishing the specific impact of each factor is challenging. Moreover, the duration of altered microbiota and its relationship with the persistence of respiratory symptoms remain unknown, warranting a longer follow-up study for clarification.

Fourth, the alteration of sputum microbiota was associated with the infected pathogen. Rhinovirus infections exhibited enrichment of *Enterococcus* and *Stenotrophomonas*, aligning with previous studies reporting coinfection of Rhinovirus with *Stenotrophomonas maltophilia* or *Enterococcus faecium* [39, 40]. Meanwhile, influenza A infections showed enrichment of *Acinetobacter* and *Pseudomonas*, indicating a possible increased susceptibility to *Acinetobacter baumannii* and *Pseudomonas aeruginosa* after infection influenza A [41–43]. The underlying mechanism may involve viral infections damaging respiratory airways and concurrently impairing both innate and acquired immune responses. This creates a favorable environment for bacterial growth, adherence, and invasion into healthy sites of the respiratory tract [44]. Besides, *Klebsiella pneumoniae* infections, which were associated with a higher incidence of severe illness, showed more deviation from HCs (more dysbiotic) compared to *Mycoplasma pneumoniae* infections, which had a lower risk of severe illness. However, it is unclear to what extent the accompanying microbiota change, in addition to the pathogen’s direct influence, affects disease progression, as most cases with *Klebsiella pneumoniae*-positivity or *Mycoplasma pneumoniae*-positivity still possessed sputum microbiotas dominated by respiratory commensal. Such analysis is constrained by a small sample size and a diverse background microbiota, which could be overcome by conducting intervention experiments in animal models.

Our study has several limitations. First, pneumonia is a lung infection caused by various pathogens, hence the samples from the lungs (e.g., biopsy, bronchoalveolar lavage fluid) are particularly valuable. However, obtaining such samples involves invasive procedures, and longitudinal sampling is challenging. While sputum is commonly used as a proxy for lung samples [45], it inevitably contains upper respiratory tract microbes. The accuracy of sputum microbiota in reflecting lung microbiota is still debated [46, 47]. Second, the healthy microbiota data were obtained from three previous studies on the Chinese population, potentially differing from the population investigated in this study. We compared the CAP microbiota to different healthy datasets and reported only consistent results, making our conclusions more robust. Third, the use of antibiotics may influence sputum microbiota during hospitalization, but controlling this confounding factor is challenging as patients were not treated following the same protocol. Therefore, our analyses primarily focused on the samples taken upon admission when limited medical intervention had been applied. Fourth, the utilization of 16 S rRNA gene sequencing restricted our study to primarily assessing the relationship between the abundance of genus-level microorganisms and the disease, while the functional attributes of the sputum microbiota were merely predicted by the bioinformatic method. Further investigations employing metagenomic and metatranscriptomic technologies are warranted to elucidate the more precise role exerted by airway microorganisms in respiratory infectious diseases.

## Conclusion

In summary, our study demonstrated diverse sputum microbiota compositions in CAP patients, with many, especially in non-severe patients, resembling those in healthy individuals. Severe CAP cases were more likely to have microbiota dominated by potentially pathogenic bacteria and underwent greater changes during hospitalization. Further studies, especially prospective and intervention studies, are needed to decipher the causality between the respiratory microbiota change and disease severity.

## Methods

### Patients and sample collection

Spontaneous sputum samples were collected on days 1, 3, 5, 7, and 9 after admission from 367 CAP inpatients from six hospitals (Tongji Hospital, The Second Affiliated Hospital of Harbin Medical University, The First Affiliated Hospital of Xi’an Jiaotong University, The Third People’s Hospital of Shenzhen, ZhongDa Hospital, Fujian Provincial Hospital) located in different cities representing distinct geographical locations in mainland China between 2014 and 2017 (Fig. 1A). Sputum quality was assessed by the presence of polymorphonuclear neutrophils (PMNs) and squamous epithelial cells (SECs) per low-power (microscopic) field (LPF) [×10 objective]. Only qualified samples (> 25 PMNs and < 10 s per LPF) were included in the study [48]. The sputum samples were immediately placed into a viral transport medium and stored at -80℃ until transported to the lab for processing (normally within a year).

Patients in this study were diagnosed with CAP through guidelines for the diagnosis and treatment of community-acquired pneumonia [49], meeting inclusion criteria included clinical manifestations of acute infection, respiratory symptoms, inflammatory changes revealed by chest X-rays or computed tomography, and no history of healthcare system exposure. In addition, the study primarily included patients who developed symptoms within 7 days. Patients who had been ill for more than 7 days and experienced a sudden worsening of symptoms during treatment, suggestive of a possible secondary infection, were also included. Cases of pneumonia caused by non-infectious factors were excluded. The severity of the patients was determined following the Guideline of the American Thoracic Society and Infectious Diseases Society of America [50]. Specifically, CAP patients must meet one primary criterion or three secondary criteria to be classified as clinically severe CAP cases. Primary criteria included 1). requirement for invasive mechanical ventilation; 2). presence of septic shock necessitating vasopressor therapy. Secondary criteria were 1). respiratory rate ≥ 30 breaths/minute; 2). PaO2/FiO2 ratio ≤ 250; 3). Multilobar infiltrates; 4). altered mental status or disorientation; 5). Renal dysfunction (blood urea nitrogen level ≥ 20 mg/dL); 6). Leukopenia (white blood cell count < 4 × 10^9/L); 7). Thrombocytopenia (platelet count < 100 × 10^9/L); 8). Hypothermia (core body temperature < 36.0 °C); 9). Hypotension requiring aggressive fluid resuscitation. Patients diagnosed with severe pneumonia at any time during hospitalization are recorded as severe cases.

### Statistical analysis

The alpha diversity was calculated by the estimate_richness function in R package phyloseq(v.1.38.0) [51]. Beta diversity represented by Jensen-Shannon Divergence (JSD) distance was calculated by Phyloseq R package (v4.0.3) [51]. Permutational multivariate analysis of variance (PERMANOVA) was used to compare the microbiota composition between different groups [52], p-value was calculated based on 999 permutations. All the possible confounders (variables 1–10 in Table 1) were used for multivariate PERMANOVA. Wilcoxon signed-rank test was used to compare continuous variables in different groups. Fisher’s exact test was used to test the correlation between categorical variables. P-values were adjusted for multiple testing using the Benjamini-Hochberg method.

Additional methods applied in the study were described in the supplementary methods.

### Electronic supplementary material

Below is the link to the electronic supplementary material.

Supplementary Material 1

Supplementary Material 2

Supplementary Material 3

## Data Availability

Raw sequencing data have been deposited in the GSA in the National Genomics Data Center (HRA002709). All statistical analyses were implemented in RStudio and the scripts and data could be accessed at https://github.com/zhanglinfeng164/CAP_sputum_microbiota.

## Abbreviations

COPD: chronic obstructive pulmonary disease
BMI: body-mass index
PERMANOVA: permutational multivariate analysis of variance
CAP: community-acquired pneumonia
NCs: negative controls
HCs: healthy controls
JSD: Jensen–Shannon divergence
PCoA: principal coordinate analysis
PERMONAVA: permutational multivariate analysis of variance
LEfSe: linear discriminant analysis effect size
LDA: linear discriminant analysis
ROC: receiver operator characteristic
CS: cluster
SpiecEasi: sparse inverse covariance estimation for ecological association inference
