# Supplementary material for: Highly diverse sputum microbiota correlates with the disease severity in patients with community-acquired pneumonia: a longitudinal cohort study
Source: Respir Res. 2024 May 29;25:223. doi: 10.1186/s12931-024-02821-2 (PMC11137881; doi:10.1186/s12931-024-02821-2)
Supplement: Supplementary file 1 — Supplementary Material 1 [file 12931_2024_2821_MOESM1_ESM.docx]

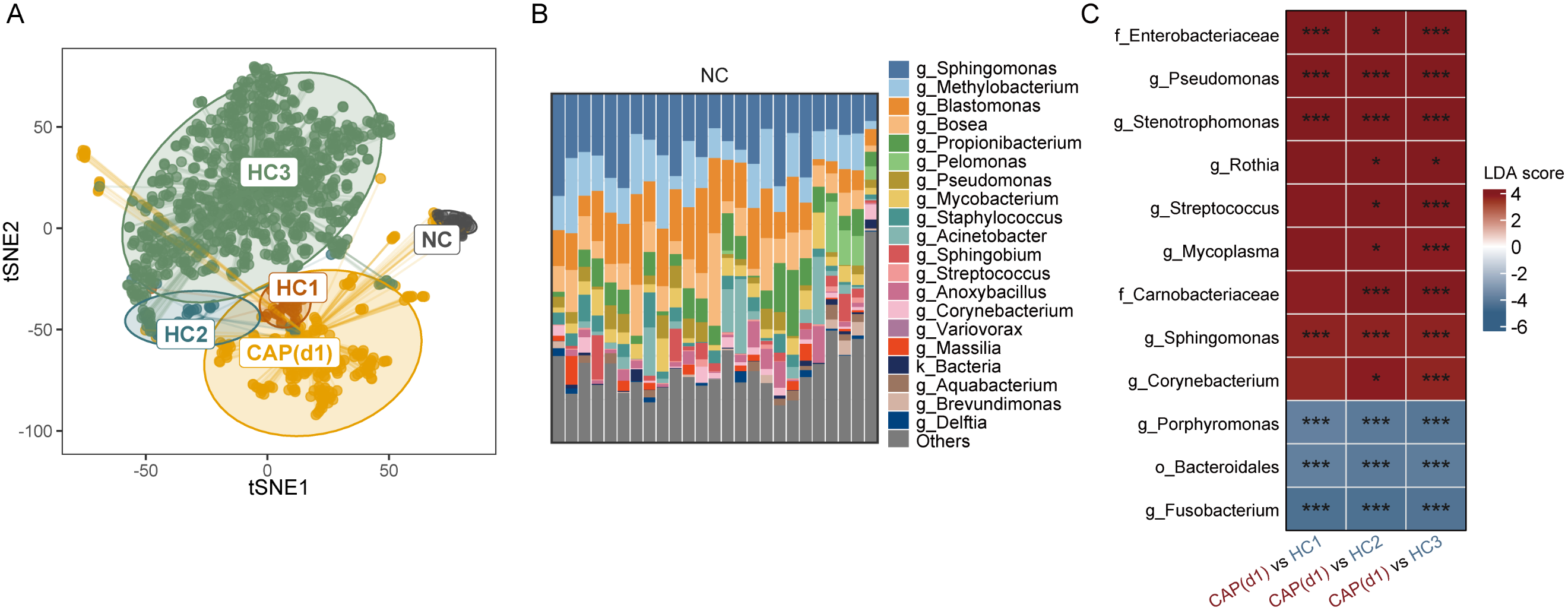


**Supplementary Figure 1 Difference in the sputum microbiota between CAP patients and healthy individuals.** (**A**). t-SNE (t-distributed stochastic neighbor embedding) plot of the microbiota from CAP patients and HCs. (**B**). Microbiota composition in the NCs. (**C**). Bacteria whose abundance differed between HCs and CAP patients identified by LEfSe (|LDA| > 4, p < 0.05). Only CAP samples collected on admission were included in the analysis. The LDA score denotes the degree of enrichment of the bacterium in samples that are labeled in red on the x-axis.

**
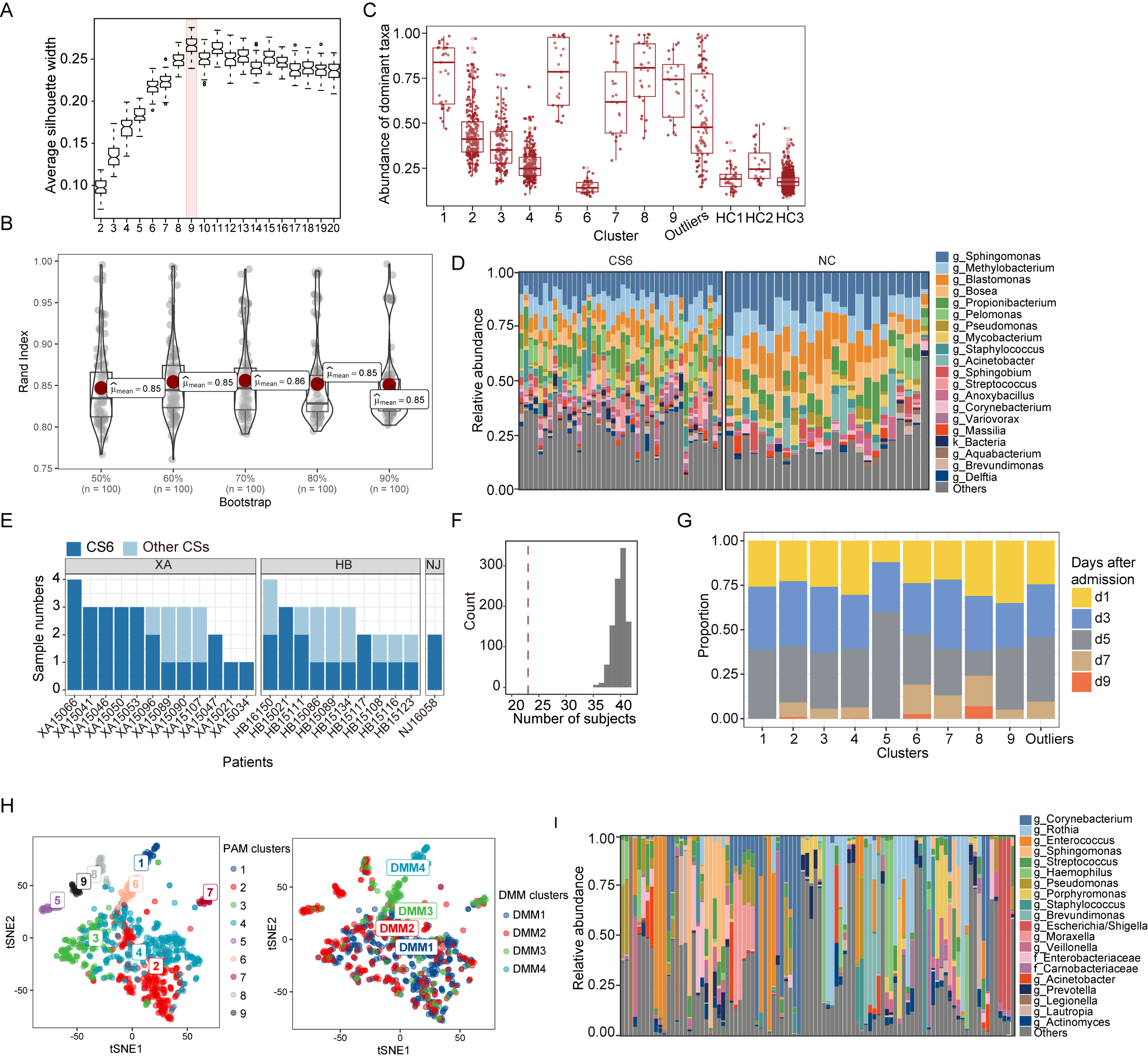
**

**Supplementary Figure 2 Features of the sputum microbiota clusters in CAP patients.** (**A**). Average silhouette index with different cluster numbers. The highest value was highlighted in pink. (**B**). Bootstrap analysis for evaluating the robustness of clusters. (**C**). The abundance of the dominant bacterium in different clusters, outlier samples (that cannot be assigned to any specific clusters), and HCs. (**D**). Microbiota composition in the NCs and samples belonging to CS6. (**E**). Distribution of different CSs in 23 CAP patients who have at least one sample assigned to CS6 (42 CS6 and 19 other CSs in total). (**F**). Expected number of patients with CS6 when 42 CS6 samples were randomly distributed in all patients. The simulation was replicated 1000 times. The red vertical line represents the observed number of patients with 42 CS6 samples. (**G**). The distribution of samples from different time points in each cluster. (**H**). t-SNE plots of the microbiota from CAP patients clustered by PAM and DMM. (**I**). Microbiota composition of outlier samples that cannot be assigned to any specific clusters.


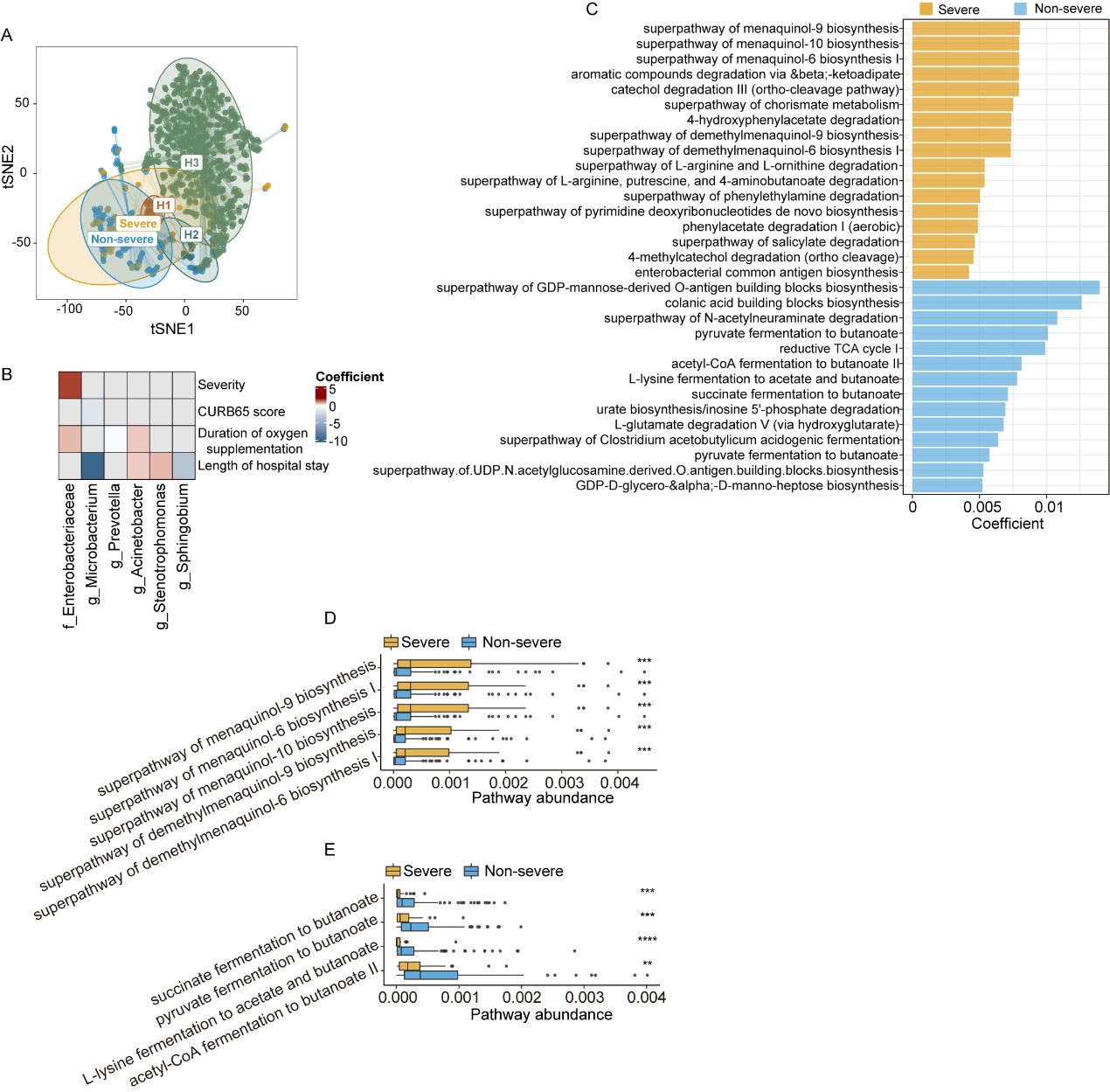


**Supplementary Figure 3 The correlation between sputum microbiota and disease severity.** (**A**). t-SNE plot of samples from severe CAP cases, non-severe CAP cases, and HCs. (**B**). Bacteria that were associated with disease severity, CURB65 scores, duration of oxygen supplementation, and length of hospital stay, were identified by MaAsLin2. Variables 1-10 in Table 1 were included as covariates in the analysis. (**C**). Pathways associated with disease severity identified by MaAsLin2. (**D**). Relative abundance of pathways belonging to "menaquinol biosynthesis pathways" and "demethylmenaquinol biosynthesis pathways" in severe and non-severe CAP cases. (E). Relative abundance of pathways belonging to "fermentation to butanoate pathways" in severe and non-severe CAP cases. * p < 0.05, ** p<0.01, *** p<0.001, ****p<=0.0001.


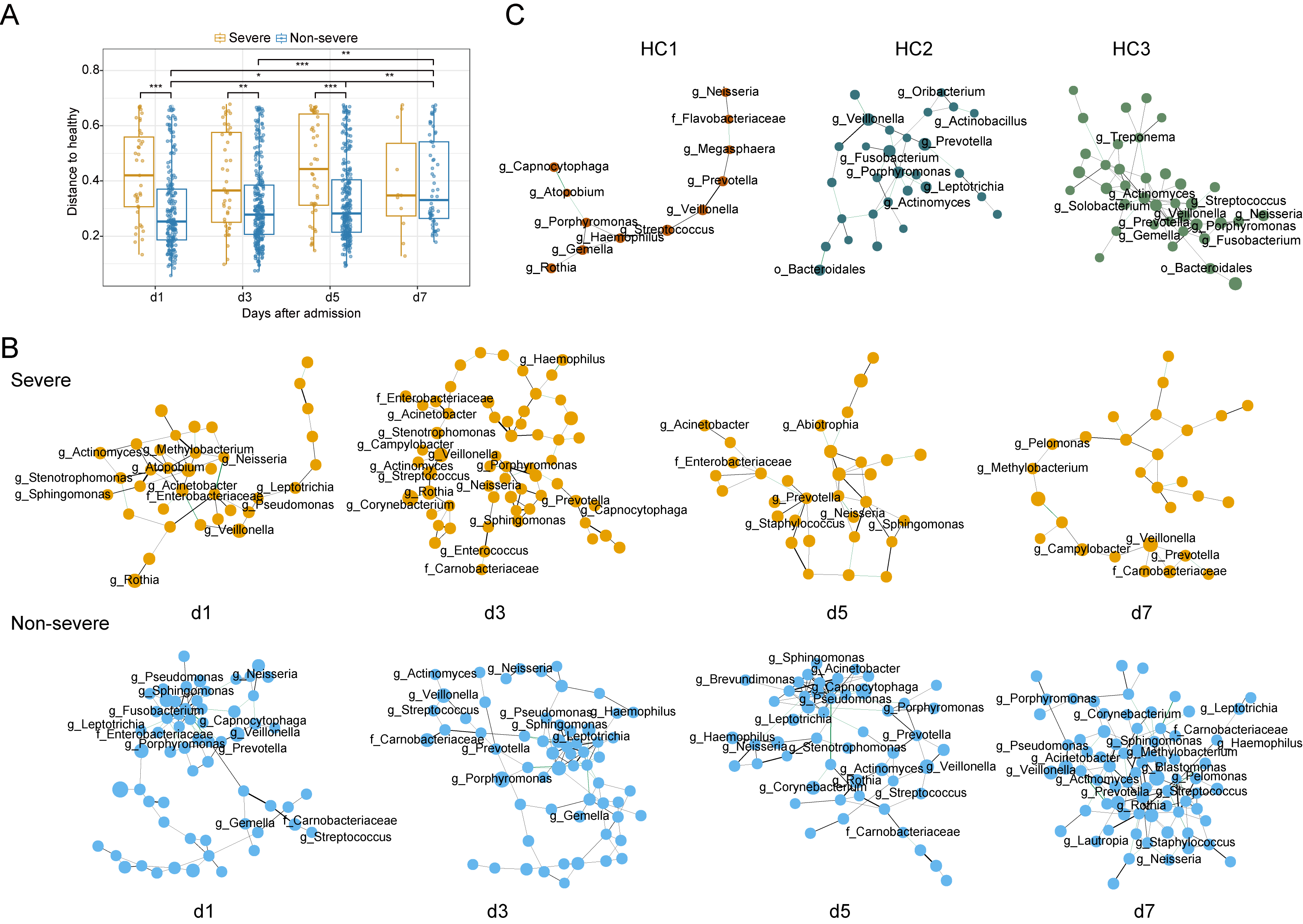


**Supplementary Figure 4 Dynamics of the sputum microbiota after admission.** **(A).** JSD distance of CAP patients to the healthy individual after admission. The data from severe patients were labeled in yellow and the data from non-severe CAP cases were labeled in blue. The microbiota composition of the three HC groups was averaged and used as the HC to calculate the distance. **(B)**. Giant component of concurrent networks constructed by SpiecEasi in severe and non-severe CAP cases at different time points. **(C)** Giant component of concurrent networks constructed by SpiecEasi in three HCs. Each node denotes a bacterial microbe. The size of nodes represents the mean abundance of microbes, and the labels of microbes with a mean relative abundance greater than 0.5% are shown in the networks. Black lines represent positive correlations between microbes while green lines represent negative correlations. The thickness of the lines denotes the magnitude of the correlation. * p.adj<0.05, ** p.adj<0.01, *** p.adj<0.001, **** p.adj<0.0001.

**
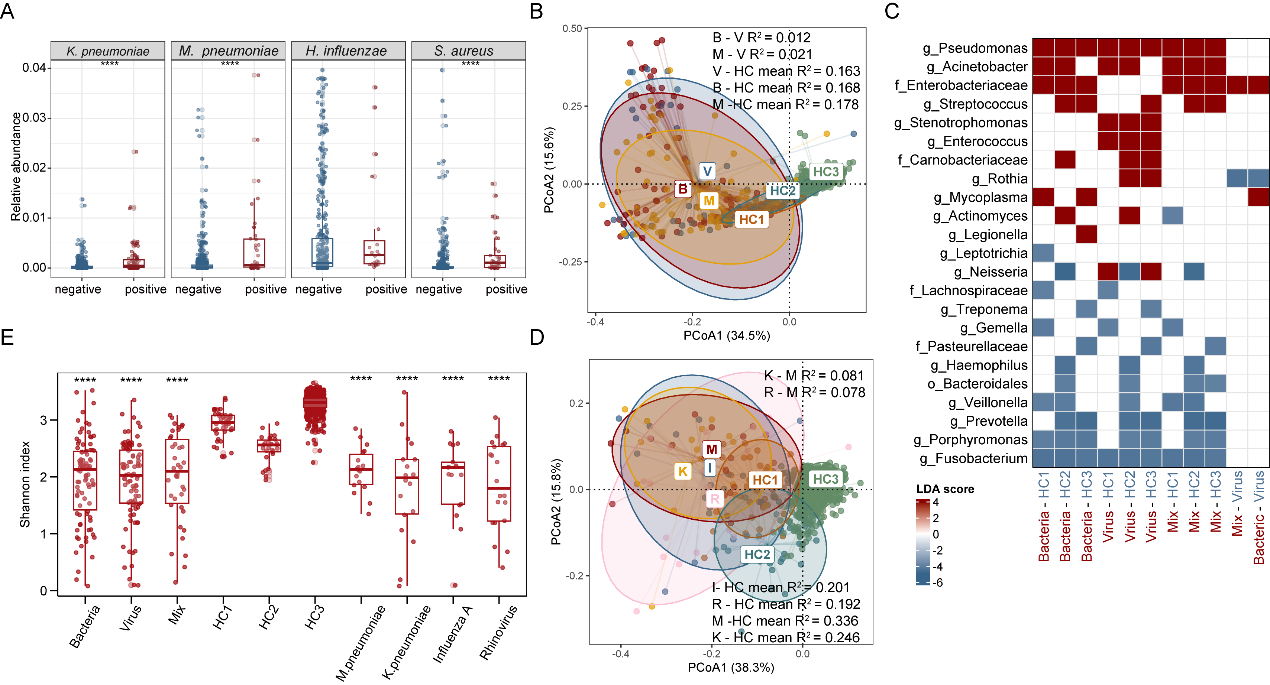
**

**Supplementary Figure 5 Comparison of the sputum microbiota between different pathogen infections.** (**A**). The abundance of bacteria identified in FTD® nucleic acids positive and negative samples. Only bacteria with FTD® nucleic acids positive results in more than 20 patients were included in the analysis. (**B**). PCoA plot of samples from patients infected by bacteria, viruses, mix (coinfected by bacterial and viral pathogens), and healthy individuals. B denotes bacterial infection, V denotes viral infection, and M denotes mixed infection. (**C**). Bacteria that were associated with different types of infections were identified by LEfSe (|LDA| 4, p<0.05). The LDA score denotes the degree of enrichment of the bacterium in the infection type that is labeled in red on the x-axis. (**D**). PCoA plot of samples from patients infected by *Mycoplasma pneumoniae*, *Klebsiella pneumonia*, Influenza A, and Rhinovirus, and healthy individuals. M denotes *Mycoplasma pneumonia* infection, K denotes *Klebsiella pneumonia* infection, and I denotes Influenza A infection, R denotes Rhinovirus infection. (E). Shannon index of samples infected by different pathogens and that from HCs. Statistical significance was determined by comparing each cluster with all the HCs. * p<0.05, ** p<0.01, *** p<0.001, **** p<0.0001.
