# Supplementary material for: Highly diverse sputum microbiota correlates with the disease severity in patients with community-acquired pneumonia: a longitudinal cohort study
Source: Respir Res. 2024 May 29;25:223. doi: 10.1186/s12931-024-02821-2 (PMC11137881; doi:10.1186/s12931-024-02821-2)
Supplement: Supplementary file 2 — Supplementary Material 2 [file 12931_2024_2821_MOESM2_ESM.docx]

**Supplementary methods**

**Nucleic acid extraction, library preparation, and sequencing**

DNA was extracted using NucliSENS® easyMAG™ (bioMérieux, France). The V3-V4 variable region of bacteria 16S rRNA was amplified using the primers with 6bp barcode, 338F (5′-NNNNNNACTCCTACGGGAGGCAGCAG -3′), 806R (5′-NNNNNNG GACTACHVGGGTWTCTAAT-3′).(1) The PCR cycling protocol consisted of pre-denaturation at 95 °C for 3 mins, 35 cycles of denaturation at 95 °C for 15s, annealing at 51 °C for 15s, extension at 72 °C for 45s, and final extension at 72 °C for 5 mins. The PCR products were purified by Agencourt AMPure XP Kit (Beckman, USA) and quantified with Qubit dsDNA HS Assay Kit (Life Technologies, USA) according to the manufacturer's instructions. Libraries for all samples were prepared by VAHTSTM Universal DNA Kit Library Prep Kit for Illumina® V2 (Vazyme, China), following the manufacturer's instructions. Libraries were sequenced on an Illumina MiSeq platform using the mode of 250-bp paired-end reads. To control for contaminations introduced during the sample processing, 28 deionized water samples that were processed following the same protocol as clinical samples were used as negative controls.

**Sequencing data processing and taxonomic assignment**

Demultiplexing and adapter and primer trimming were performed by Cutadapt (v2.6).(2) Human reads were removed by aligning reads to GRCH38 by BMTagger (v3.101-3). Filtration, merge of paired-end reads, ASV construction, and removal of chimera were executed by DADA2 module implemented in QIIME2 (v2019.10) (denoise-paired, --p-chimera-method 'consensus' --p-max-ee-f 2 --p-max-ee-r 3.3).(3, 4) Taxonomy of ASV was assigned by USEARCH (v11.0.667, SINTAX algorithm) using the adjusted Ribosomal Database Project (RDP) training set v16 (cutoff = 0.8). The reference tree was built by SEPP fragment-insertion algorithm implemented in QIIME2.(5, 6) The ASV table was rarefied to 10000 reads, and species with relative abundance greater than 0.01 in at least one sample were used for subsequent analysis. Prediction of functional pathways was performed by PICRUST2.(7) Sputum microbiota data of 953 healthy individuals were retrieved from three previous studies (Table S2) and analyzed following the same pipeline,(8-10) resulting in the successful profiling of microbiota from 876 individuals.

**Clustering of the microbiota data**

Two methods, the partition around medoids (PAM) algorithm and Dirichlet Mixture Model (DMM),(11) were applied for clustering. For the former method, Silhouette Index was used to estimate the number of clustering, and nine clusters were recommended by the method (Fig. S2A), whereas four clusters were recommended by the DMM method based on the lowest Laplace approximation score. We noted that the intra-cluster heterogeneity was much higher for the DMM clusters, with microbiota dominated by different bacteria being assigned to the same cluster (Fig. S2H). Thus, we decided to adhere to the clusters assigned by the PAM method. Moreover, the robustness of PAM clusters was validated by a bootstrap analysis. We sub-sampled 50%, 60%, 70%, 80%, and 90% of the total 917 samples 100 times, respectively, and re-clustered each subset of samples into nine clusters by PAM method. Then, we used the Rand Index (RI), a metric of measuring the agreement between two sets of clusters, to quantify the similarity of the resulting clusters from each subset of samples to the clusters with all the samples, which provided a robust mean RI of around 0.85. Notably, the samples whose microbiota compositions significantly differed from other samples in the same cluster were defined as outliers (Bray-Curtis distance > 0.75).

**Differential analysis**

Linear discriminant analysis effect size (LEfSe)(12) and Microbiome Multivariable Association with Linear Models (MaAsLin2, v1.4.0)(13) were applied to identify the bacteria that were enriched in a specific group. In MaAsLin2, variables 1-10 in Table 1 were used as covariates (Harbin, which showed the largest deviation from other cities in terms of microbiota composition, was used as the reference city). The abundance of the bacteria was converted using the acsin function, and a zero-adjusted model CPLM (Compound Poisson) was chose to apply.

The L1 regularized logistic regression model was used to identify the bacteria that could distinguish severe CAP from non-severe CAP, the AUC-ROC was used to evaluate the performance of the classifier. The analysis was conducted using the Scikit-learn Python package.(14)

**Definition of pathogenic and core microbes**

Generalist bacteria that were observed in more than 70% of respiratory samples in 13 cohorts were defined as core respiratory bacteria,(15) and the non-core bacteria that were included in the pathogen list provided by CZID (<https://czid.org/pathogen_list>) were defined as possible pathogenic pathogens. For bacteria that were assigned to a higher taxonomic level, e.g. family, they were defined as pathogenic if any genus belonging to the taxon was included in the pathogen list. A list of all possbile pathogenic and core bacteria identified in our study was included in Table S2.

**Co-occurrence network analysis**

The co-occurrent network was calculated by the spiec.easi function in R package SpiecEasi(v.1.1.2).(16) Only bacteria shared by at least 20% of samples in each group were considered in the analysis. The giant component of the co-occurrence network, which contained the highest number of nodes, was chosen to represent the network by giant_component_extract function in (1.2.0).(17)

**Respiratory pathogens screening**

The nucleic acids were extracted from respiratory samples and were used for known common respiratory pathogens screening by a multiplex quantitative polymerase chain reaction (PCR) test (FTD® Respiratory Pathogen 33 assay)(Fast Track Diagnostics, Luxembourg) , including influenza A virus, influenza B virus, influenza C virus, IFVA/2009/H1N1, human parainfluenza viruses 1, 2, 3 and 4, human coronaviruses NL63, 229E, OC43, and HKU1, human metapneumoviruses A and B, human rhinoviruses, human respiratory syncytial viruses A and B, human adenovirus, enteroviruses, human parechovirus, human bocavirus, *Pneumocystis jirovecii*, *Mycoplasma pneumoniae*, *Chlamydophila pneumoniae*, *Streptococcus pneumoniae*, *Haemophilus influenzae*, *Haemophilus influenzae type B*, *Staphylococcus aureus*, *Moraxella catarrhalis*, *Bordetella* spp (except for *Bordetella parapertussis*), *Klebsiella pneumoniae*, *Legionella pneumophila*, and *Salmonella* spp.

**Reference**

1. Mancabelli L, Milani C, Lugli GA, Turroni F, Cocconi D, van Sinderen D, et al. Identification of universal gut microbial biomarkers of common human intestinal diseases by meta-analysis. FEMS Microbiol Ecol. 2017;93(12).

2. Martin M. CUTADAPT removes adapter sequences from high-throughput sequencing reads. EMBnetjournal. 2011;17.

3. Bolyen E, Rideout JR, Dillon MR, Bokulich NA, Abnet CC, Al-Ghalith GA, et al. Reproducible, interactive, scalable and extensible microbiome data science using QIIME 2. Nat Biotechnol. 2019;37(8):852-7.

4. Callahan BJ, McMurdie PJ, Rosen MJ, Han AW, Johnson AJA, Holmes SP. DADA2: High-resolution sample inference from Illumina amplicon data. Nat Methods. 2016;13(7):581-3.

5. Janssen S, McDonald D, Gonzalez A, Navas-Molina JA, Jiang L, Xu ZZ, et al. Phylogenetic Placement of Exact Amplicon Sequences Improves Associations with Clinical Information. mSystems. 2018;3(3).

6. Edgar RC, Flyvbjerg H. Error filtering, pair assembly and error correction for next-generation sequencing reads. Bioinformatics. 2015;31(21):3476-82.

7. Douglas GM, Maffei VJ, Zaneveld JR, Yurgel SN, Brown JR, Taylor CM, et al. PICRUSt2 for prediction of metagenome functions. Nat Biotechnol. 2020;38(6):685-8.

8. Du S, Shang L, Zou X, Deng X, Sun A, Mu S, et al. Azithromycin Exposure Induces Transient Microbial Composition Shifts and Decreases the Airway Microbiota Resilience from Outdoor PM(2.5) Stress in Healthy Adults: a Randomized, Double-Blind, Placebo-Controlled Trial. Microbiology spectrum. 2023;11(3):e0206622.

9. Cai X, Luo Y, Zhang Y, Lin Y, Wu B, Cao Z, et al. Airway microecology in rifampicin-resistant and rifampicin-sensitive pulmonary tuberculosis patients. BMC Microbiol. 2022;22(1):286.

10. Lin L, Yi X, Liu H, Meng R, Li S, Liu X, et al. The airway microbiome mediates the interaction between environmental exposure and respiratory health in humans. Nat Med. 2023;29(7):1750-9.

11. Pal S, Heumann C. Clustering compositional data using Dirichlet mixture model. PloS one. 2022;17(5):e0268438.

12. Segata N, Izard J, Waldron L, Gevers D, Miropolsky L, Garrett WS, et al. Metagenomic biomarker discovery and explanation. Genome Biology. 2011;12:R60 - R.

13. Mallick H, Rahnavard A, McIver LJ, Ma S, Zhang Y, Nguyen LH, et al. Multivariable Association Discovery in Population-scale Meta-omics Studies. bioRxiv. 2021:2021.01.20.427420.

14. Pedregosa F, Varoquaux G, Gramfort A, Michel V, Thirion B, Grisel O, et al. Scikit-learn: Machine Learning in Python. Journal of Machine Learning Research. 2012;12.

15. Einarsson GG, Zhao J, LiPuma JJ, Downey DG, Tunney MM, Elborn JS. Community analysis and co-occurrence patterns in airway microbial communities during health and disease. ERJ Open Res. 2019;5(3).

16. Kurtz ZD, Müller CL, Miraldi ER, Littman DR, Blaser MJ, Bonneau RA. Sparse and compositionally robust inference of microbial ecological networks. PLoS Comput Biol. 2015;11(5):e1004226.

17. Ashtiani M, Mirzaie M, Jafari M. CINNA: an R/CRAN package to decipher Central Informative Nodes in Network Analysis. Bioinformatics. 2019;35(8):1436-7.
